# Proton Therapy for Malignant Pleural Mesothelioma: A Three Case Series Describing the Clinical and Dosimetric Advantages of Proton-Based Therapy

**DOI:** 10.7759/cureus.1705

**Published:** 2017-09-20

**Authors:** Howard Lee, Jing Zeng, Stephen R Bowen, Ramesh Rengan

**Affiliations:** 1 School of Medicine, Duke University School of Medicine; 2 Radiation Oncology, University of Washington School of Medicine

**Keywords:** mesothelioma, malignant pleural mesothelioma, proton, radiation pneumonitis, pneumonectomy, radiation, radiation oncology, intensity modulated proton therapy (impt), intensity modulated radiotherapy (imrt)

## Abstract

Malignant pleural mesothelioma (MPM) is a malignancy of the pleural cavity that typically presents at an advanced stage. Due to its large, circumferential clinical target volume (CTV) and proximity to major structures, including the heart and contralateral lung, delivering hemithoracic intensity-modulated radiotherapy (IMRT) with photon therapy to achieve loco-regional control following macroscopic complete resection is challenging. Intensity-modulated proton therapy (IMPT) has been shown to be a method for achieving higher therapeutic doses while limiting exposure to organs at risk (OARs), but patient outcomes after treatment have yet to be reported. We present three patients who received IMPT to 54 Gy after extrapleural pneumonectomy (EPP), with two patients receiving boosts to 66 and 60 Gy. All three tolerated treatment well and received doses to OARs markedly lower than those seen in comparison volumetric-modulated arc therapy (VMAT) IMRT photon plans. Radiation pneumonitis, a highly morbid and potentially fatal toxicity in patients receiving thoracic radiotherapy, was not observed even with boost treatments. In practice, IMPT appears to match dosimetric predictions as a feasible and safer alternative to photon IMRT-based radiotherapy.

## Introduction

In the treatment of malignant pleural mesothelioma (MPM), radiotherapy is often administered to 50-54 Gray following neoadjuvant chemotherapy and extrapleural pneumonectomy (EPP) [1]. However, a potential hazard in current photon intensity-modulated radiotherapy (IMRT) -based plans is the dose to critical organs at risk (OARs), including the heart, spinal cord, and especially the contralateral lung, which is associated with clinically significant pneumonitis [2]. A higher mean lung dose (MLD) and the volume of lung receiving 5, 10, or 20 Gy (V5, V10, V20) are associated with greater risks for toxicity [3]. Intensity-modulated proton therapy (IMPT), with its rapid dose falloff at pre-determinable treatment depths, is thus a promising modality for escalating doses to complex target volumes, such as lung-encasing mesotheliomas, while sparing OARs. However, the long-term outcomes and implications of patients undergoing such treatments have yet to be reported. Here, we report three cases of patients treated post-EPP with IMPT.

Treatment techniques included pencil beam scanning (PBS) delivery using single field optimization (SFO) inverse planning. Planning target volume (PTV) margins were 5 mm and clinical target volume (CTV) margins were 5 mm - 8 mm. Target doses were normalized such that 95% of the PTV received at least 95% of the prescription. Plans were evaluated for the robustness of CTV dose coverage under range and density (three percent), then setup uncertainty (+/- 3 mm isotropic nominal). These uncertainties take into account the density changes in a lung target and the associated water equivalent path length for each proton beam. Two to four beams were used combining anterior oblique and posterior orientations, with greater weight (up to 70%) given to posterior beams due to reduced dosimetric uncertainty.

## Case presentation

Case one

A 71-year-old man was diagnosed with Stage III (pT3N2) biphasic MPM. The level 3A, level 7, and level 8 mediastinal nodes were positive. The patient received neoadjuvant cisplatin/pemetrexed and underwent right-sided EPP. The tumor involved the posterior, middle, and inferior visceral, parietal, and diaphragmatic pleurae. Surgical margins were close but negative. Fifty-four Gy was delivered in 30 fractions to the postoperative bed and the mediastinal region by IMPT, with a simultaneous boost to 66 Gy to specific areas at the highest risk of residual disease indicated in the operative note. The most significant adverse effects were common terminology criteria for adverse events (CTCAE) version four grade two nausea and dermatitis.

By four months post-radiation, the patient was experiencing dyspnea on exertion, and a computed tomography (CT) scan demonstrated very small, indeterminate nodules in the contralateral lung. He elected for observation, and by a nine months' interval, the growth of these nodules indicated recurrence. The patient continued to have dyspnea on exertion and a dry cough but strongly preferred not to pursue additional treatment at that time. By the 18-month follow-up, the patient was having increased cough and right-sided posterior chest pain as well as abdominal discomfort attributed to disease recurrence. At the 20-month follow-up, the patient started pembrolizumab, and by 22 months, was experiencing stable dyspnea on exertion with moderate to strenuous effort, as well as a stable cough and a persistent right-sided stabbing chest pain. After a repeat CT scan demonstrated tumor progression while on treatment, he transitioned to hospice and passed away at 25 months post-radiation.

Case two

A 47-year-old man was diagnosed with right-sided Stage IV (pT4N2) epithelioid MPM. One 4R mediastinal node was positive. His tumor showed poor response to neoadjuvant cisplatin/pemetrexed but pre-operative positron emission tomography-CT (PET-CT) did not show any gross extension of disease into the chest wall and diaphragm. However, during right-sided EPP, the tumor was found to have a diffuse involvement of the pleural space and multiple positive margins, with the tumor extending into the chest wall and diaphragmatic fat in the costovertebral angle. It also exhibited trans-diaphragmatic extension with an involvement of the peritoneum and abutment of the pericardium.

Given the diffusely positive nature of the margins and the lack of any specific regions with higher risk, the patient received hemithoracic IMPT to 54 Gy. He experienced grade one nausea and fatigue and grade two dermatitis. A CT scan three months post-radiation demonstrated new soft tissue thickening of the right costovertebral angle and new subcentimeter hepatic hypodensities indicating distant recurrences of the disease. The patient pursued palliative care and passed away four months post-radiation.

Case three

A 46-year-old man was diagnosed with right-sided Stage III (pT3N0) epithelioid, node-negative MPM. He received cisplatin/pemetrexed and right-sided EPP. Pathology showed evidence of mediastinal fat invasion and multiple microscopically positive margins.

The patient received hemithoracic IMPT to 54 Gy with a simultaneous boost to 60 Gy to high-risk areas described in the operative note. He tolerated treatment well, experiencing grade one dermatitis, cough, and nausea. A CT chest at seven weeks post-radiation showed no evidence of a recurrent intrathoracic disease or radiation-associated changes. By his five-month follow-up, the patient was doing well with good energy and weight gain and was able to walk one mile. He continued to have an occasional dry cough and some incisional pain. The cough completely resolved by his eight-month follow-up, but the patient was referred to physical therapy for continued right-sided chest pain near the incision. The patient is now 14 months post-radiation with no radiographic evidence of progression. His right-sided chest pain remains both stable and tolerable and is managed with physical therapy and pain medication.

## Discussion

To the best of our knowledge, this case series is the first describing patient outcomes of adjuvant IMPT in the management of MPM following EPP. Although a previous study has demonstrated their experience of the dosimetric advantage of IMPT over photon IMRT in this setting [4], we provide clinical follow-up data alongside our dosimetric experience for this rare indication.

Dosimetry

The dosimetric advantage of IMPT for thoracic malignancies has been addressed in previous publications [5], and comparison plans suggest that IMPT allows markedly better OAR sparing compared to photon IMRT, mainly for the liver, ipsilateral kidney, heart, and, most importantly, the contralateral lung [6]. There seems to be a dose-effect relationship for fatal pneumonitis, and photon-based IMRT appears to be safest when the mean lung dose to the contralateral lung is ≤8.5 Gy [7].

In our three patients treated to 54 Gy with or without boosts of up to 66 Gy, we were able to achieve contralateral mean lung doses of 1.53, 0.74, and 0.26 Gy and without the development of radiation pneumonitis. The mean dose delivered to the PTV, boost dose, and each organ at risk, including selected percent volumes of each OAR receiving 5-50 Gy, are shown in Table 1. These values for IMPT are compared to corresponding values extracted from volumetric-modulated arc therapy (VMAT) IMRT photon-based plans generated on the same patients and normalized to the same target coverage constraints. Our IMPT OAR dose goals were also applied to photon VMAT plan optimization; for example, contralateral lung dose goals were between MLD 5-9 Gy, V20 10-35%, V10 10-50%, and V5 15-65%. Immediately evident is that VMAT plans with the same prescription dose and target coverage would not be clinically approved. For example, the VMAT comparison plan generated for Patient One with the same prescription, boost, and target coverage as the IMPT plan shows that contralateral lung MLD = 18.5 Gy, V5 = 78.2%, and V20 = 18.9%. VMAT plans generated to meet clinical constraints of contralateral lung MLD < 8 Gy, V5 < 60%, and V20 <7% (<10% max) would be required to sacrifice dose or target coverage. This highlights the benefit of proton therapy in the ability to dose escalate while preserving target coverage and achieving acceptable doses to OARs. 

**Table 1:** Summary of Dose Volume Histogram Analysis for PTV and OARs Mean doses in Gy, volumes of OARs receiving 5-50 Gy in % - comparison of intensity-modulated proton therapy (IMPT) treatment plans to volumetric-modulated arc therapy (VMAT) “full dose” plans generated on the same patients utilizing the same target constraints. PTV: planning target volume, OARs: ​​​​organs at risk.

	PTV	Boost	Contra Lung (mean dose, Gy)	V5 (%)	V10 (%)	V20 (%)	Heart (mean dose, Gy)	V5 (%)	V30 (%)	V40 (%)	Cord D1 (%)	Liver (mean dose, Gy)	V30 (%)	Ipsil Kidney (mean dose, Gy)	V20 (%)	Esoph (mean dose, Gy)	V50 (%)
Proton one	54	66	1.5	8.5	4.9	1.9	4.6	20.7	3.6	1.6	41.9	25.3	40.5	38.1	97.7	30.8	37.1
VMAT one	54	66	18.5	78.2	52.5	18.9	17.4	98.3	13.8	6.5	41.1	30.0	44.2	47.5	100	35.7	37.3
Proton two	54	0	0.7	4.1	2.1	0.5	14.2	39.9	23.3	18.3	46.0	17.1	28.7	15.4	30.9	31.3	12.4
VMAT two	54	0	12.9	99.7	56.0	15.9	27.9	100	37.3	29.0	49.3	39.2	70.1	17.0	29.2	39.8	25.0
Proton three	54	60	0.3	1.1	0.4	0.1	11.1	29.6	17.4	14.3	44.4	25.5	33.5	12.4	22.7	30.2	25.0
VMAT three	54	60	13.5	89.3	58.3	19.3	26.2	100	29.4	22.8	50.2	25.3	34.1	14.3	27.9	39.0	36.1

Compared to photon-based VMAT IMRT, conventional step-and-shoot photon IMRT would be expected to deliver a reduced low-dose bath to the surrounding structures given necessary fixed-angle beam pre-selection. VMAT, with its arc-based therapy, allows highly conformal treatment for irregular tumor volumes, but with multiple dose angles, has the disadvantage of this low-dose bath. This may be relevant when treating lung-encasing mesotheliomas surrounded by parenchyma prone to radiation pneumonitis. Helical tomotherapy, with its multiple overlapping helical arc-based treatments, would be expected to deliver slightly more conformal treatment than VMAT but at the expense of increased radiation delivery time, the clinical effect of which is still largely unknown. Tomotherapy is also subject to a low-dose bath like VMAT. However, none of these photon IMRT-based treatment modalities would be expected to achieve dosimetric constraints similar to IMPT. 

In addition to a comparison with VMAT plans generated for the same patients with the same target coverages, we also compared our dosimetric parameters with conventional photon IMRT values calculated at the Brescia University Hospital, Italy, for seven patients receiving 50 Gy, three of whom received boosts to 60 Gy [6]. Even with lower prescription and boost doses than our IMPT patients, the photon IMRT patients in the Brescia study received significantly higher doses to every OAR. For example, the average V5, V20, and MLD for the contralateral lung were 39.1%, 5.8%, and 6.1 Gy, respectively in the Brescia study. In comparison, the average V5, V20, and MLD for the contralateral lung in our IMPT experience were 3.4%, 2.5%, and 0.8 Gy, respectively.

We consider the contralateral lung a critical OAR given the risk of fatal pneumonitis. Brigham and Women’s Hospital reported this rate to be as high as 46% in its treatment of 13 patients [8]. Patients developing fatal pneumonitis had a median V5, V20, and MLD of 98.6%, 17.6%, and 15.2 Gy, respectively, while patients not developing pneumonitis had a median V5, V20, and MLD of 90%, 10.9%, and 12.9 Gy, respectively.

Duke University Hospital has also reported its photon IMRT experience, and patients receiving a median prescription dose of 45 Gy, with an average V5 of 56%, V20 of 4%, and contralateral mean lung dose of 7.2 Gy went on to develop grade three or higher radiation pneumonitis in 13% of the cases [9].

**Figure 1:**
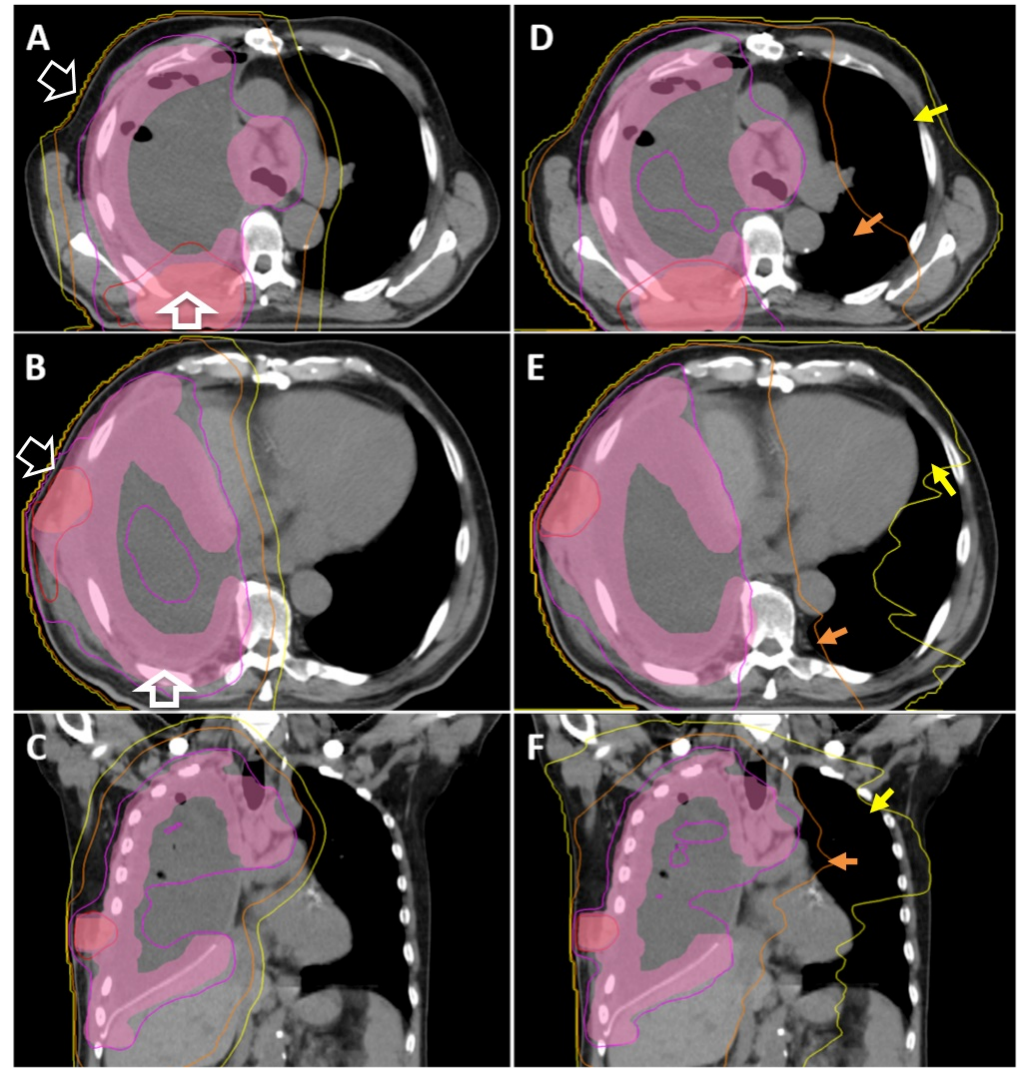
IMPT Treatment Plan versus Photon VMAT Comparison Plan for Patient One IMPT (A-C) isodose lines and shaded planning target volume (PTV) contours versus those of a VMAT (D-F) comparison plan for Patient One. Red line = 6270 cGy, Magenta line = 5130 cGy, Orange line = 2000 cGy, Yellow line = 500 cGy. Light red shaded contour = PTV boosted to 64 Gy. Fuchsia shaded contour = PTV treated to 54 Gy. The volume of contralateral lung receiving above 5 Gy is 8.45% in the IMPT plan (A-C), and 78.2% in the VMAT plan (D-F). Yellow arrows highlight regions of contralateral lung receiving above 5 Gy in the VMAT plan but not in the corresponding regions of the IMPT plan. The volume of contralateral lung receiving above 20 Gy is 1.9% in the IMPT plan (A-C) and 18.9% in the VMAT plan (D-F). Orange arrows highlight regions of contralateral lung receiving above 20 Gy in the VMAT plan but not in the corresponding regions of the IMPT plan. White arrows indicate anterior oblique and posterior to anterior proton beam angles. Greater line width for the posterior to anterior beam indicates preferential weight given up to 70%, as it is dosimetrically more robust to proton range uncertainties. IMPT: intensity-modulated proton therapy. VMAT: volumetric-modulated arc therapy.

Figure 1 visually demonstrates the degree to which our case with the highest dose, Patient One, received lower doses to volumes of contralateral lung in IMPT treatment versus a comparison VMAT plan (V5 = 8.45% vs 78.2%, respectively). Overall, our patients treated with IMPT received such reduced doses to the contralateral lung and other OARs compared to those in VMAT and other photon IMRT-based plans that OAR-based toxicity should be a significantly reduced concern.

Toxicities

Our patients completed treatment without breaks and experienced radiation-induced nausea and dermatitis at grades no worse than photon IMRT patients treated to 48-54 Gy [10]. Although Patient One developed dyspnea on exertion and Patient Three developed a mild nonproductive cough, none of our patients had corresponding fevers or imaging findings suggesting radiation-induced pneumonitis, such as ground-glass attenuation or patchy areas of consolidation along treatment fields for acute (four to 12 weeks) or subacute (three to six months) pneumonitis. Patient One’s dyspnea on exertion at four months is better explained by a progression of the disease. Patient Three’s non-productive cough that had resolved by eight months could be due to a subacute radiation pneumonitis, but given the patient’s lack of fevers or radiographic findings, we would favor other etiologies such as laryngeal irritation due to structural shifts secondary to pneumonectomy.

## Conclusions

The management of MPM is challenging, and techniques for utilizing IMPT to improve postoperative loco-regional control have been proposed. To our knowledge, this is the first report documenting both dosimetric and clinical outcomes of adjuvant IMPT in MPM patients post-EPP. Our series is limited by the small numbers and relatively short follow-up for our patients, and we are unable to perform a survival comparison with larger published series. Based on our experience with a maximum follow-up of 25 months and a median follow-up of 14 months, doses of up to 66 Gy appear feasible and safe. OAR dose profiles are significantly lower than those seen in photon VMAT and IMRT plans, especially for the contralateral lung and heart, theoretically greatly decreasing the likelihood of OAR-based toxicities. Photon VMAT and IMRT plans that achieve the same prescription dose and target coverage as IMPT plans cannot be employed clinically given the unacceptable dose to OARs. 

## References

[1] Chen SE, Pace MB (2012). Malignant pleural mesothelioma. Am J Health Syst Pharm.

[2] Miles EF, Larrier NA, Kelsey CR, Hubbs JL, Ma J, Yoo S, Marks LB (2008). Intensity-modulated radiotherapy for resected mesothelioma: the Duke experience. Int J Radiat Oncol Biol Phys.

[3] Rice DC, Smythe WR, Liao Z (2007). Dose-dependent pulmonary toxicity after postoperative intensity-modulated radiotherapy for malignant pleural mesothelioma. Int J Radiat Oncol Biol Phys.

[4] Pan HY, Jiang S, Sutton J (2015). Early experience with intensity modulated proton therapy for lung-intact mesothelioma: a case series. Pract Radiat Oncol.

[5] Chang JY, Li H, Zhu XR (2014). Clinical implementation of intensity modulated proton therapy for thoracic malignancies. Int J Radiat Oncol Biol Phys.

[6] Lorentini S, Amichetti M, Spiazzi L, Tonoli S, Magrini SM, Fellin F, Schwarz M (2012). Adjuvant intensity-modulated proton therapy in malignant pleural mesothelioma. A comparison with intensity-modulated radiotherapy and a spot size variation assessment. Strahlenther Onkol.

[7] Pinto C, Ardizzoni A, Betta PG (2011). Expert opinions of the first Italian consensus conference on the management of malignant pleural mesothelioma. Am J Clin Oncol.

[8] Allen AM, Czerminska M, Janne PA (2006). Fatal pneumonitis associated with intensity-modulated radiation therapy for mesothelioma. Int J Radiat Oncol Biol Phys.

[9] Patel PR, Yoo S, Broadwater G (2012). Effect of increasing experience on dosimetric and clinical outcomes in the management of malignant pleural mesothelioma with intensity-modulated radiation therapy. Int J Radiat Oncol Biol Phys.

[10] Thieke C, Nicolay NH, Sterzing F (2015). Long-term results in malignant pleural mesothelioma treated with neoadjuvant chemotherapy, extrapleural pneumonectomy and intensity-modulated radiotherapy. Radiat Oncol.

